# Effect of live‐high, train‐low strategy induced by chronic low‐dose carbon monoxide exposure on haematological parameters and performance in trained individuals

**DOI:** 10.1113/EP093005

**Published:** 2026-01-12

**Authors:** Simone Villanova, Simone Porcelli, Lena Ekström, Daniele A. Cardinale

**Affiliations:** ^1^ Department of Movement, Human and Health Sciences University of Rome ‘Foro Italico’ Rome Italy; ^2^ Department of Molecular Medicine University of Pavia Pavia Italy; ^3^ Department of Laboratory Medicine Division of Clinical Pharmacology Karolinska Institutet Stockholm Sweden; ^4^ Department of Physiology, Nutrition and Biomechanics The Swedish School of Sport and Health Sciences GIH Stockholm Sweden; ^5^ Department of Physiology and Pharmacology Karolinska Institutet Stockholm Sweden

**Keywords:** elite athlete, endurance performance, erythropoiesis, haemoglobin mass, hypoxia, mitochondria

## Abstract

Altitude training enhances haematological adaptations and endurance at sea level, typically requiring exposure to ∼2500 m altitude for 3–4 weeks. Emerging evidence suggests that low‐dose carbon monoxide (CO) inhalation might mimic hypoxia and might be used by elite athletes. In this study, we examine whether periodic low‐dose CO exposure can replicate the live‐high, train‐low model in well‐trained individuals, focusing primarily on haematological and performance effects of CO exposure, with haematological markers commonly used to interpret haemoglobin mass changes discussed as exploratory. Eight well‐trained individuals (four males and four females) participated in a randomized crossover study. They completed two training blocks of 4 weeks at sea level: one with CO inhalation (INCO) to simulate live‐high, train‐low and one with ambient air as a control (AIR), separated by a 6 month washout. Haematological variables, in vivo muscle oxidative capacity and performance metrics were assessed before and after each intervention. After INCO, haemoglobin mass (*p* = 0.018; +53.6 ± 10.8 g. vs. +0.8 ± 11.8 g), red blood cell volume (*p* = 0.032; +156.6 ± 66.7 mL vs. −65.1 ± 50.7 mL) and blood volume (*p* = 0.036; +240.4 ± 120.5 mL vs. −208.3 ± 167.5 mL) increased significantly compared with AIR. INCO significantly reduced immature reticulocytes (*p* = 0.04), but muscle oxidative capacity and performance metrics remained unchanged. These findings suggest that daily low‐dose CO exposure at sea level over 4 weeks enhanced haematological adaptations more than standard training but did not affect muscle oxidative capacity or performance.

## Abstract (highlights)

- **What is the central question of this study?**
  We investigated whether chronic, low‐dose carbon monoxide inhalation at sea level can replicate the haematological adaptations typically achieved through the live‐high, train‐low altitude training model in well‐trained individuals.
- **What is the main finding and its importance?**
  We found that daily low‐dose carbon monoxide inhalation over 4 weeks significantly increased haemoglobin mass and red blood cell volume, mimicking altitude‐induced haematological adaptations, without improving muscle oxidative capacity or performance. No changes in other haematological parameters classically used to interpret changes in haemoglobin mass were observed.

## 1 INTRODUCTION

Periodic inhalation of carbon monoxide (CO) at rest has been shown to mimic the effects of hypoxic training (Wang et al., 2019) and living at high altitude (Schmidt et al., 2020), with additive benefits when combined with natural high‐altitude exposure (Urianstad et al., 2024). This approach challenges traditional altitude training models and, given that the use of CO specifically to replicate the ‘live‐high, train‐low’ (LHTL) strategy remains underexplored, merits investigation. Notably, there are indications that elite athletes might already be using, or misusing, CO as a performance‐enhancing method (Minson & Joyner, 2024).

Within the current landscape of hypoxic training (Girard et al., 2020) and terrestrial or simulated altitude‐exposure strategies (Millet et al., 2010), the LHTL strategy combines the benefits of altitude acclimatization, such as enhanced oxygen (O_2_) transport and improved muscle metabolism, without compromising exercise training intensity (Levine & Stray‐Gundersen, 1997). These characteristics make LHTL a recognized practice (Girard et al., 2023). However, it is not necessarily a superior strategy in comparison to traditional altitude training for preparing athletes to compete at high altitude and to improve athletic performance at sea level (Bonetti & Hopkins, 2009).

Irrespective of the hypoxic strategy adopted, a reduction in arterial O_2_ content, induced by either a reduction in the partial pressure of O_2_ (hypobaric hypoxia) or a direct reduction of O_2_ concentration (normobaric hypoxia), triggers the activation of hypoxia‐inducible factor‐1α (Semenza, 2000), subsequently leading to an increased erythropoietic response (Montero & Lundby, 2019) and rising haemoglobin mass (Hb_mass_) within a few weeks (Chapman et al., 1998). Besides, altitude training can also increase skeletal muscle buffer capacity and exercise economy (Garvican et al., 2011; Gore et al., 2001), potentially contributing to improved athletic performance. Based on a meta‐analysis of altitude‐training studies accounting for both the duration and the severity of hypoxic exposure (Garvican‐Lewis et al., 2016), an accumulated hypoxic dose of ∼1000 km h can lead to a ∼4% increase in Hb_mass_, which is approximately twice the typical measurement error. The ‘km h’ metric is calculated by multiplying the altitude in kilometres by the total number of exposure hours (e.g., 2100 m × 0.001 × 504 h = 1058 km h). This dose could be achieved through various protocols, for instance as 21 days at 2100 m a.s.l. using the traditional LHTH model or as 21 days at 3000 m a.s.l. for 16 h days using the LHTL approach. Notably, increases in Hb_mass_ are possible even in elite athletes with high baseline values, provided the hypoxic dose is sufficient (Millet et al., 2019).

However, logistical constraints, race schedule and the limited access to appropriate high‐altitude facilities (Constantini et al., 2017) often lead athletes to short camps at relatively low altitudes. In such cases, longer exposure durations are required to provide an adequate adaptive hypoxic dose. Importantly, a meta‐analysis by Gore et al. (2013) found a median Hb_mass_ increase of 3.52% following a standardized 300 h hypoxic exposure (≥2100 m a.s.l.), with 95% of individuals exhibiting true responses between 1.14% and 5.95%. This wide variability underscores the importance of individual responsiveness in the design and evaluation of altitude training programmes. Emerging evidence (Schmidt et al., 2020; Urianstad et al., 2024) suggests that carbon monoxide (CO) inhalation mimics hypoxic conditions (Richardson et al., 2002) by reducing functional haemoglobin availability (Douglas et al., 1912; Haldane, 1912), while stimulating erythropoiesis (Montero & Lundby, 2019) without causing hypoxic‐induced tachycardia and hypoxic ventilatory response (Hanada et al., 2003).

Carbon monoxide is an endogenous molecule typically present in blood as carboxyhaemoglobin at concentrations [COHb] of <2% in non‐smokers. In smokers, [COHb] values between 5% and 13% are reported (Rosenthal, 2006), and heavy smokers can display [COHb] of ∼20% (Sen et al., 2010). CO has a half‐life of 4–5 h based on ventilation, and acute exposures that increase [COHb] up to 10%–15% are typically not associated with side effects (Foresti et al., 2008) even during near‐maximal exercise (Ekblom & Huot, 1972).

Moreover, CO acts as a signalling molecule, promoting anti‐inflammatory responses (Zuckerbraun et al., 2007), mitochondrial biogenesis (Rhodes et al., 2009; Suliman et al., 2007) and angiogenesis (Pecorella et al., 2015), along with other beneficial cardiovascular functions (for a review on this topic, see Durante et al., 2006). However, it remains unclear whether CO uptake occurs in skeletal muscle tissue (Chance et al., 1970; Hogan et al., 1990; Richardson et al., 2002).

A previous study (Schmidt et al., 2020) demonstrated that administering six daily doses of CO, maintaining [COHb] at 4%–7% throughout the day over 3 weeks, effectively simulating a live‐high, train‐high approach, resulted in a 4.8% increase in Hb_mass_, which was correlated with a similar improvement in maximal oxygen consumption (${\dot{V}}_{O_{2}\max}$) in healthy, moderately trained men. A more recent study (Urianstad et al., 2024) indicated that CO supplementation had a positive additive effect on haematological parameters when combined with moderate altitude training, in comparison to both the live‐high, train‐high approach alone and sea‐level training.

To explore the effects of administration of low doses of CO on haematological adaptations and exercise performance further, we designed a 4 week crossover training study, in which participants were blindly exposed to a low dose of CO or ambient air, and the effects of CO on Hb_mass_ and intravascular blood volumes, skeletal muscle oxidative capacity and performance in well‐trained individuals were assessed.

Until recently, the use of CO was not a method specifically prohibited by World Anti‐Doping Agency (WADA). However, the 2026 Prohibited List now explicitly bans the use of rebreathing systems or equipment to deliver CO, unless performed as a diagnostic procedure under medical or scientific supervision (WADA, 2025). Concerns had already been raised regarding CO inhalation among athletes, and a direct ban of repeated CO use has been suggested (Minson & Joyner, 2024) following the study by Urianstad et al. (2024). However, it would be challenging to monitor CO exposure (Faiss & Krumm, 2024). Given that haematological parameters, including [Hb] and reticulocyte count, are monitored in an Athlete Biological Passport to detect blood doping (Sottas et al., 2011), as a secondary aim of this study we also explored the effects of CO supplementation on these markers to interpret Hb_mass_ changes better. The hypothesis was that exposure of individuals living at sea level to low doses of CO at rest, simulating the LHTL approach, would enhance Hb_mass_ and O_2_ delivery to skeletal muscle, thus enhancing muscle oxidative capacity, leading to improvements in submaximal and maximal swimming performance, in comparison to training at sea level as the control conditions.

## 2 MATERIALS AND METHODS

### 2.1 Ethical approval

The study followed the ethical standards of the *Declaration of Helsinki*, except for registration in a database. It was approved by the Swedish Ethical Review Authority (2022‐05697‐02). Before participating, all participants received both verbal and written information about the study design and protocol and provided written informed consent.

### 2.2 Study design

Each participant completed two swimming training blocks, each lasting 4 weeks, except for one swimmer who performed a 3 week block for reasons not associated with the study. In a single‐blind, randomized design, participants were assigned to either the treatment with CO inhalation (INCO) to mimic the ‘live‐high, train‐low’ approach or the ‘sea‐level control’ approach, inhaling ambient air as a placebo (AIR). After a washout period of ∼6 months, participants completed the second training block in the alternative conditions. Both training blocks were preceded by a transition period of 3–4 weeks characterized by reduced training volume and several rest days. One week before the beginning of each 4 week training block, participants were tested for body composition, haematological assessment, blood lactate profile in swimming, muscle oxidative capacity by near‐infrared spectroscopy (NIRS) and swimming performance. The same testing protocol was completed at the end of each 4 week block.

### 2.3 Study participants

Four female (height 1.79 ± 0.09 m, body mass 75.8 ± 5.6 kg and age 25.5 ± 5.4 years) and four male (height 1.92 ± 0.12 m, body mass 85.9 ± 10.5 kg and age 25.0 ± 4.1 years) well‐trained individuals participated in the study.

As a precaution against potential negative effects of iron deficiency, the daily intake of 25 or 100 mg of oral iron supplement (AminoJern, Ferrochel, Albion Laboratories Inc., USA or Duroferon Aco Hud Nordic AB, Sweden) was recommended to all participants based on individual ferritin levels starting 1 week before the first day of the intervention and continuing until the post‐testing was completed (Govus et al., 2015; Okazaki et al., 2019). Based on the typical error of Hb_mass_ measurement and an expected minimum change of 2% in Hb_mass_ between the two training blocks, at least six participants were required in this cross‐over study design to achieve 80% power and a 5% significance level.

### 2.4 Anthropometrics and body composition

The body composition characteristics of participants were measured with a dual‐energy X‐ray absorptiometry scan (model Horizon A, S/N 305579 M) in an overnight fasting state, with participants wearing underwear and lying in a supine position on the examination bed.

### 2.5 Haemoglobin mass and intravascular blood volumes

Participants were tested for total Hb_mass_ and intravascular blood volumes using a CO‐rebreathing procedure, which was either 2 or 6 min in duration. The same procedure was performed for each participant both before and after each training block.

The 2 min CO‐rebreathing procedure has been described in detail elsewhere (Schmidt & Prommer, 2005). Briefly, with the participants sitting on a chair, 5 mL of blood was sampled from an antecubital vein via a 20‐gauge Venflon and analysed immediately for haemoglobin concentration ([Hb]), [COHb] and haematocrit (HCT). After baseline collection, the end‐tidal CO concentration of participants was measured with a portable CO detector (Dräger Pac 6500; Drägerwerk AG, Lübeck, Germany) before proceeding with the breathing procedure. The procedure was initiated by the participant wearing a nose‐clip, initially exhaling through the mouth to near residual volume. Thereafter, the participant inhaled an individualized bolus (1 or 1.2 mL kg^−1^ body mass for women and men, respectively) of chemically pure CO (CO N47, Air Liquide, Paris, France) injected into the circuit via a prefilled 100 mL syringe while deeply inhaling pure oxygen (Air Liquide, Paris, France) through a mouthpiece connected to a spirometer and the oxygen reservoir. After this first inspiration, the participant held their breath for 10 s, then continued normal respiration from the spirometer for 1 min 50 s. The participant was then disconnected from the spirometer after exhaling residual volume. Four minutes from the start of the test, the end‐tidal CO concentration was again measured with the portable CO detector. At minute 6 after CO inhalation, an additional venous blood sample was collected from an antecubital vein for assessment of the change in [COHb], accounting for the CO remaining in the rebreathing circuit, which was determined (Monoxor III, Bacharach Inc., New Kensington, PA, USA). The Hb_mass_ was calculated from the change in [COHb], and the total red blood cell volume (RBCV), blood volume (BV) and plasma volume (PV) were derived (Burge & Skinner, 1995).

The 6 min CO‐rebreathing procedure has been described in detail elsewhere (Siebenmann et al., 2017) and involved the use of a semi‐automated blood volume analyser (OpCO, Detalo Performance, Detalo Health, Birkerød, Denmark), which is validated (Breenfeldt Andersen et al., 2023). Briefly, participants rested for ∼15 min lying down in a semi‐recumbent position. A blood sample was obtained from the fingertip (45 µL) and immediately analysed for [Hb], [COHb] and HCT, followed by breathing 100% oxygen (Airliquide, Paris, France) for 15–30 s. Subsequently, the participants rebreathed a bolus of chemically pure CO (CO N47, Air Liquide, Paris, France) corresponding to 1.0 mL kg^−1^ for 6 min. During the rebreathing period, oxygen was administrated on a demand basis. The amount of CO not absorbed by the participants during the rebreathing period was determined by automatic assessments of the volume of the system multiplied by the parts per million of CO remaining in the system after the rebreathing period (Dräger Pac 6500; Drägerwerk AG, Lübeck, Germany). Directly after terminating the rebreathing, a second blood sample was obtained from the fingertip (45 µL) and immediately analysed in triplicate for [COHb]. All blood values were entered into the device software, where Hb_mass_, RBCV, total BV and PV were calculated. The Hb_mass_ was also expressed relative to individual body mass (relative Hb_mass_). Following a 5 min rest, the entire rebreathing procedure was repeated to obtain duplicate measurements for all study participants. Duplicate measurements differed in Hb_mass_ on average by 1.8%, with a typical error (calculated as the SD of the differences between duplicates divided by √2) of 19.7 g.

Independently of the rebreathing method, the [COHb] and [Hb] were measured in triplicate with an haemoximeter using a spectrophotometric method (ABL800Flex, Radiometer, Copenhagen, Denmark), whereas HCT was measured in triplicate using the microhaematocrit method after centrifuging the capillaries for 3 min at 17 g‐force, 13 500 r.p.m. (Micro Star 21/21R Microcentrifuges, VWR International AB Stockholm, Sweden).

### 2.6 Haematological and hormonal profile

EDTA samples were transported at room temperature to one of the WADA accredited Doping Control Laboratories either the same day within 5 h after collection or stored at 4°C for <48 h before transportation. After arriving at the laboratory, the samples were analysed immediately with a Sysmex XN‐1000. The Sysmex XN‐1000 was used for the analyses of hemoglobin, red blood cell (RBC) count, mean corpuscular volume (MCV), mean corpuscular haemoglobin (MCH), mean corpuscular haemoglobin concentration (MCHC), percentage of reticulocytes (RET%), absolute reticulocyte count (RET#) and immature reticulocyte fraction (IRF). The OFF‐score was calculated as ([Hb] × 10) – (60 × RET%)].

The urinary steroid profile, including testosterone (T), epitestosterone (EpiT), androsterone (A), etiocholanolone (Etio), 5α‐androstane‐3α,17β‐diol (5αAdiol) and 5β‐androstane‐3α,17β‐diol (5βAdiol), was analysed by gas chromatography–tandem mass spectrometry as previously described (Mullen et al., 2020). Specific gravity was measured using a digital urine SG Refractometer (ATAGO UG1, Tokyo Japan) to correct for different urine dilutions using formula: Conc_corr_ = Conc_Measured_ × (1.020 – 1)/[(SG + 0.002) – 1] where Con_ccorr_ is the corrected analyte concentration and SG is the specific gravity.

### 2.7 Muscle oxygen uptake recovery rate constant by NIRS

The muscle oxidative capacity of triceps brachii was estimated non‐invasively as oxygen recovery rate constant (*k*) by NIRS while participants lay in a supine position on an examination bed. Briefly, a wireless portable continuous‐wave NIRS device (Train.Red FYER, The Netherlands) was placed on the participant's skin in correspondence to the triceps brachii muscle, and changes in tissue saturation index (TSI) were sampled at 10 Hz using the spatially resolved spectroscopy approach (Ferrari et al., 2011). A 13 cm × 85 cm rapid‐inflation pressure cuff (SC12D; Hokanson, Bellevue, WA, USA) was placed proximally on the same arm and attached to an electronically controlled rapid cuff‐inflator (E20; Hokanson). After a few minutes of rest, a prolonged arterial occlusion (∼300 mmHg) was induced until TSI reached a plateau. Immediately after, the cuff was instantly deflated, and muscle reoxygenation was recorded. The physiological range of TSI values [i.e. physiological normalization (PN)] was defined between the deflection point (minimum TSI) and the maximum value reached during the reperfusion phase (maximum TSI) (Adami et al., 2017). Then, subjects were asked to extend their elbow against a fixed resistance to reduce TSI up to 50% of PN, and a series of arterial occlusions were performed in the recovery from exercise by rapidly inflating and deflating the pressure cuff. The downslope phase of TSI changes (as a percentage per second) during each intermittent occlusion was fitted against time to establish the exponential recovery rate constant (*k*; per minute) of muscle O_2_ consumption. The investigator modulated the duration and timing of the repeated occlusions to ensure that the intermittent occlusion occurred in conditions of well‐oxygenated muscle (i.e., TSI of >50% of PN) (Pilotto et al., 2022). The coefficient of variation for repeated measurements was 18%, indicating a good reliability of the measurement.

### 2.8 Blood lactate profile and performance test

Each swimmer completed a series of performance tests in a 50 m pool, consisting of two 400 m and two 200 m trials, all performed using the front crawl technique (Olbrecht et al., 1992). The two 400 m tests were conducted at different intensities: the first at a low‐intensity speed and the second at a medium‐intensity speed within the moderate domain of exercise intensity. Likewise, the two 200 m tests were performed with the first at a speed in the heavy domain and the second at volitional maximal speed. Swimmers were instructed to maintain a steady pace during each trial. All tests began with a push start in the water.

Capillary blood samples (20 µL) were collected from the earlobe and analysed for blood lactate concentrations [La] (Biosen C‐line, EKF Diagnostics, Germany). Samples were obtained pre‐test, at 1 min post‐test and every 2 min thereafter until maximum [La] was reached.

### 2.9 Carbon monoxide administration

The participants inhaled a bolus of CO (carbon monoxide N47, Airliquide, Sweden) three times a day at rest for 6 days a week via a 100 mL syringe. The first administration occurred at ∼10.00 h, directly after the first training session of the day; the second administration occurred at 16.00–17.00 h, directly after the second training session of the day, with the third administration at ∼21.00–22.00 h. The volume of CO was inhaled directly with a single‐breath manoeuvre followed by a 30 s breath hold. The volume of each inspired CO bolus was tailored to raise individual [COHb] to 15%. Given that the total Hb_mass_ of the subjects was known, the required millilitres of CO to achieve a target 15% [COHb] were calculated using Equation (1) (Ekblom & Huot, 1972):

(1) $$\left[\left(Hb_{\mathrm{mass}}\times 1.34\right)\times 10/100\right]\times 25\%$$

For the dosage of the second bolus, the volume of the CO bolus was calculated using Equation (2)  (Schmidt et al., 2020):

(2) $$\begin{array}{ccc} \mathrm{CO}-\mathrm{Vo}l_{\mathrm{adm}} & = & {\left[\mathrm{COHb}\right]}_{t}-\left({\left[\mathrm{COHb}\right]}_{\mathrm{act}}-{\left[\mathrm{COHb}\right]}_{i}\right)\\ & & \times \;\mathrm{CO}-\mathrm{Vo}l_{i}/\Delta {\left[\mathrm{COHb}\right]}_{i} \end{array}$$

where CO‐Vol_adm_ is the CO volume to administer; [COHb]_t_ is the target COHb concentration, i.e., 10%; [COHb]_act_ is the [COHb] before inhaling the second CO bolus; [COHb]_i_ is the initial [COHb] at baseline with no prior CO inspired; CO‐Vol_i_ is the CO volume administered at the initial Hb_mass_ test; and Δ[COHb]_i_ is the difference in [COHb] at the initial Hb_mass_ test. The [COHb] post‐inhalation was checked during the intervention period using real‐time capillary blood gas analysis (ABL800 FLEX; Radiometer, Copenhagen, Danmark), and the volume of CO administered was adjusted if necessary. The mean [COHb], along with the lower and upper bounds of the 95% confidence interval, was 9.0% [1.9, 16.0] for INCO and 1.1% [1.0, 1.2] for AIR.

Although translating [COHb] levels into a simulated altitude is challenging, an estimation was made by integrating the reduction in ${\dot{V}}_{O_{2}\max}$ caused by elevated [COHb] levels (Ekblom & Huot, 1972) and factoring in a ${\dot{V}}_{O_{2}\max}$ decline of 7% for every 1000 m a.s.l. (Wehrlin & Hallen, 2006). The hypoxic dose was calculated in ‘kilometre hours’ (Garvican‐Lewis et al., 2016) using the formula:

$$kmh=(m\times 1000^{-1})\times h$$

where **m** represents the elevation of exposure in metres (e.g., 2000 m a.s.l.) and **h** is the total duration of exposure in hours (e.g., 24 h). The hypoxic dose for the INCO treatment was estimated to be 847.9 km h during the 4 week training block. In comparison, living 16 h per day for 21 days in Sierra Nevada (2320 m a.s.l.) would result in a hypoxic dose of 779.5 km h.

### 2.10 Data analysis and statistics

Normal distribution of the data was checked visually through Q–Q plots and by assessing skewness. Baseline anthropometrics and characteristics of each group were summarized using descriptive statistics. For treatment effect analyses, analysis of covariance was used, with the baseline‐test result as a covariate and the post‐test as the dependent variable. Student's paired *t*‐test was used to assess within‐block differences in pre‐ to post‐measurement. A two‐tailed *p *< 0.05 was considered significant. Inferential statements about differences in change scores between AIR and INCO were made based on univariate ANOVA. All analyses were performed using the IBM SPSS v.29 statistical package for Windows.

## 3 RESULTS

### 3.1 Training volume and intensity comparisons during the two intervention periods and body composition

Total training volume and swimming intensity distribution at low, medium and high intensities were not different between the two 4 week training blocks (Table 1). Body mass varied within ±2% from pre‐ to post‐INCO and AIR, and there was no effect of time or treatment. INCO showed a numerical increase in lean body mass (1.8%, no significant effect of time, *p* = 0.55) and a significant reduction in fat percentage (−9.1%, effect of time, *p* = 0.02). Likewise, AIR resulted in a numerical increase in lean body mass (2.9%, no significant effect of time, *p* = 0.75) and a numerical reduction in fat percentage (−7.5%, no significant effect of time, *p* = 0.05). However, there was no effect of treatment on lean body mass (*p* = 0.56) and fat percentage (*p* = 0.93).

**TABLE 1:** Training data for the two 4 week training blocks performed following the ‘live‐high, train‐low’ approach with carbon monoxide inhalation (INCO; *n* = 7) and ‘sea‐level control’ approach (AIR; *n* = 7).

Training	AIR	INCO	INCO vs. AIR
Low, km	111.5 ± 26.4	109.5 ± 20.1	0.705
Medium, km	4.4 ± 4.3	9.0 ± 8.3	0.203
High, km	4.1 ± 1.7	4.3 ± 0.7	0.762
Total volume, km	120.0 ± 28.6	122.8 ± 19.9	0.647

*Note*: Descriptive data are the mean (SD) number of kilometres swum in low‐, medium‐ and high‐intensity exercise. The *p*‐values are presented, indicating no difference between the two training periods (INCO vs. AIR).

### 3.2 Effects of INCO on haemoglobin mass and intravascular blood volumes

**FIGURE 1:**
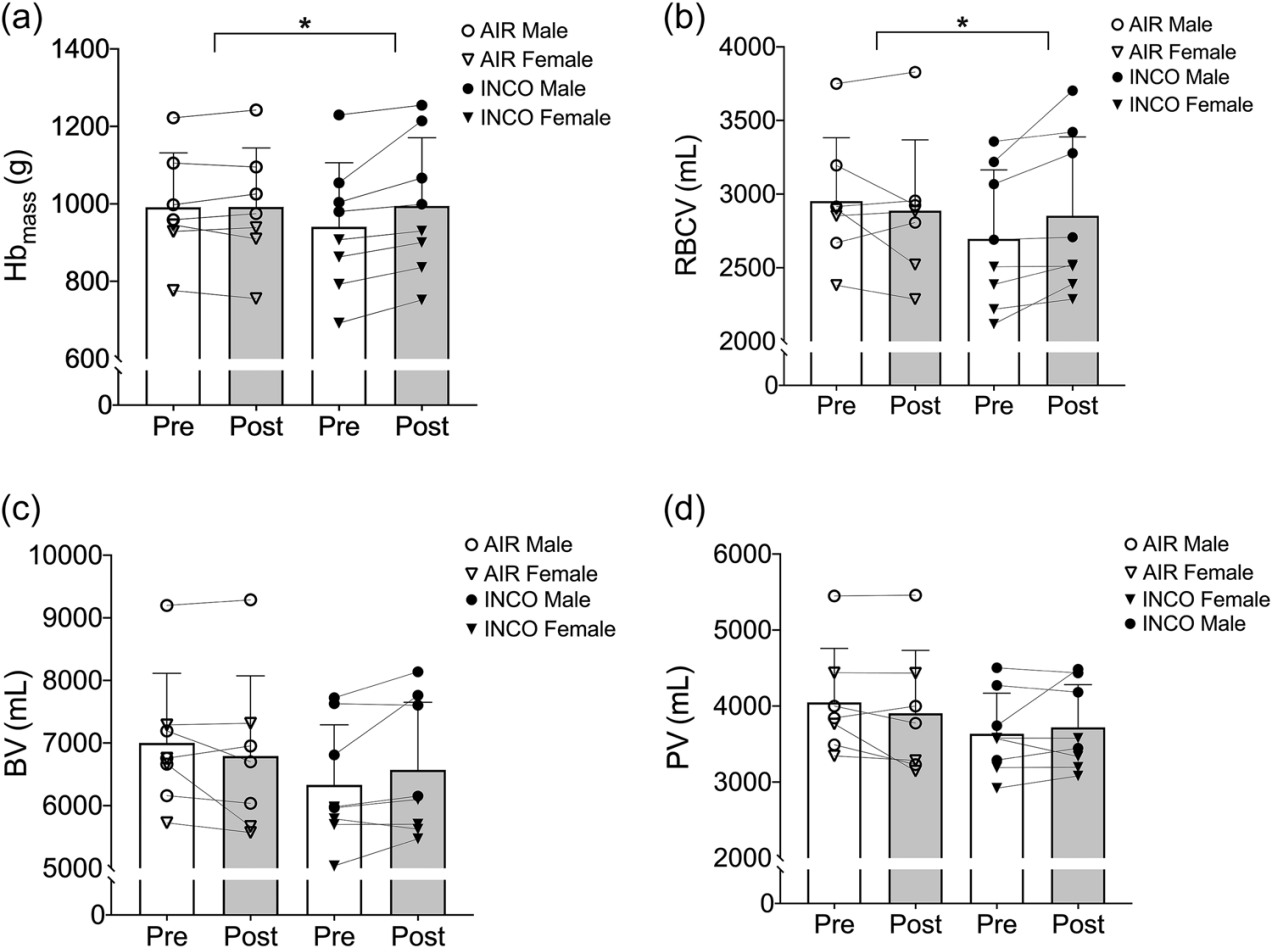
Column bar charts showing the mean + SD and individual data points (circle, male; and triangle, female) following the ‘live‐high, train‐low’ approach with carbon monoxide inhalation (INCO; *n* = 8) and the ‘sea‐level control’ approach (AIR; *n* = 7) for haemoglobin mass (Hb_mass_; a), red blood cell volume (RBCV; b), plasma volume (PV; c) and total blood volume (BV; d). *Differences between the two training periods (INCO vs. AIR).

There was a significant (time effect, *p* = 0.013) 5.7% (53.6 g) increase in total Hb_mass_ following INCO compared with AIR (treatment effect, *p* = 0.018) that showed a negligible change of 0.1% (0.8 g). For changes in [Hb] and HCT there were no significant treatment and time effects following INCO or AIR (Figure 1).

There was a significant 5.8% increase in RBCV following INCO (time effect, *p* = 0.029) compared with AIR (treatment effect, *p* = 0.032) that showed a reduction of −2.2%. The BV differed between INCO and AIR (treatment effect, *p* = 0.036), with an increase in BV of 3.8% post‐INCO and a −3.0% reduction in BV post‐AIR. The PV was increased by 2.3% post‐INCO and reduced by −3.5% post‐AIR, but there was no difference between INCO and AIR (Figure 1 and Table 2).

### 3.3 Effects of INCO on haematological and hormonal markers

No time or treatment effects were observed for the blood variables [Hb], HCT, MCH, MCHC, MCV, RBC and RET# (Table 3), except for IRF, which decreased significantly post‐INCO (time effect, *p* = 0.031) and was significantly different following AIR (treatment effect, *p* = 0.042).

There was no effect of CO on measured androgen hormone levels. However, epitestosterone, testosterone and 5α‐androstane‐3α,17β‐diol showed a significant reduction from pre‐ to post‐INCO. Additionally, epitestosterone, testosterone, the testosterone/epitestosterone ratio, 5α‐androstane‐3α,17β‐diol and 5β‐androstane‐3α,17β‐diol levels also decreased significantly post‐AIR.

### 3.4 Effects of INCO on muscle oxygen uptake recovery rate constant

No time or treatment effects were observed in the muscle oxygen uptake recovery rate following INCO and AIR (Table 2).

**TABLE 2:** Body composition, haematological variables, muscle oxygen uptake recovery and data from the submaximal and maximal swimming tests before (Pre) and after (Post) 4 week training intervention performed following the ‘live‐high, train‐low’ approach with carbon monoxide inhalation (INCO) and ‘sea‐level control’ approach (AIR).

	AIR				INCO				INCO vs. AIR
Parameter	Pre	Post	*n*	Effect of time	Pre	Post	*n*	Effect of time	
Body composition									
Body mass, kg	80.9 ± 9.5	82.3 ± 11.0	7	0.696	82.0 ± 12.4	81.2 ± 12.2	8	0.250	0.279
Lean body mass, kg	60.5 ± 8.8	62.3 ± 9.9	6	0.757	60.5 ± 9.4	61.6 ± 10.7	6	0.555	0.556
Body fat, %	17.8 ± 3.7	16.4 ± 4.4	6	0.054	18.1 ± 3.2	16.5 ± 3.4	6	0.022	0.928
Haematological variables									
[Hb], g × L^−1^	156.2 ± 6.8	158.5 ± 7.8	7	0.266	156.4 ± 10.6	159.5 ± 8.4	8	0.340	0.784
HCT, %	46.9 ± 2.8	46.3 ± 1.9	7	0.605	45.1 ± 4.6	46.0 ± 4.5	8	0.348	0.498
Hb_mass_, g	990.7 ± 141.2	991.5 ± 153.0	7	0.929	940.4 ± 165.9	994.0 ± 176.7	8	0.013	0.018
Hb_mass_, g × kg^−1^	12.1 ± 0.7	12.3 ± 0.9	7	0.386	11.6 ± 1.1	12.0 ± 1.2	8	0.122	0.377
RBCV, mL	2951.4 ± 431.4	2886.3 ± 482.1	7	0.405	2695.3 ± 469.3	2851.9 ± 536.1	8	0.029	0.032
PV, mL	4046.5 ± 712.3	3903.0 ± 830.7	7	0.186	3633.2 ± 535.8	3717.0 ± 565.4	8	0.450	0.154
BV, mL	6997.9 ± 1115.0	6789.6 ± 1282.4	7	0.230	6328.7 ± 961.9	6569.1 ± 1082.5	8	0.097	0.036
Muscle oxygen uptake									
*k* high, min^−1^	2.9 ± 0.4	3.1 ± 0.7	6	0.457	3.0 ± 0.5	3.0 ± 0.5	7	0.297	0.237
Submaximal exercise test									
Speed at 4 mM [La], m × s^−1^	1.4 ± 0.1	1.4 ± 0.1	6	0.352	1.4 ± 0.1	1.4 ± 0.0	6	0.947	0.612
Speed at 2 mM [La]. m × s^−1^	1.3 ± 0.1	1.3 ± 0.1	6	0.597	1.3 ± 0.1	1.3 ± 0.1	6	0.855	0.884
Maximal exercise test									
Speed 200 m, m × s^−1^	1.5 ± 0.1	1.5 ± 0.1	6	0.886	1.5 ± 0.1	1.5 ± 0.1	6	0.376	0.500
[La]_max_, mM × L^−1^	11.6 ± 3.2	12.2 ± 3.4	6	0.607	11.2 ± 2.5	12.8 ± 4.2	6	0.157	0.386
HR_max_, beats × min^−1^	186.0 ± 7.2	188.8 ± 5.2	6	0.204	186.0 ± 6.9	183.5 ± 6.3	6	0.595	0.125

*Note*: Descriptive data are the mean (SD) for the following parameters: BV, total blood volume; [Hb], haemoglobin concentration; Hb_mass_, haemoglobin mass; HCT, haematocrit; *k*, muscle oxygen uptake recovery constant obtained in high tissue oxygenation; PV, plasma volume; RBCV, red blood cell volume; Speed 2 and 4 mM, swimming speed at 2 and 4 mmol L^−1^ blood lactate [La]; Speed 200 m, mean swimming speed during 200 swimming test; mM L^−1^ [blood lactate]; HR_max_, maximal heart rate (in beats per minute) recorded during the 200 m swimming test. The P‐values are reported indicating the difference between pre and post each training block (effect of time) and between the two training periods (INCO vs. AIR).

**TABLE 3:** Haematological and hormonal profile before (Pre) and after (Post) 4 week training intervention performed following the ‘live‐high, train‐low’ approach with carbon monoxide inhalation (INCO) and the ‘sea‐level control’ approach (AIR).

	AIR				INCO				INCO vs AIR
Parameter	Pre	Post	*n*	Effect of time	Pre	Post	*n*	Effect of time	
IRF, %	11.1 ± 7.4	12.6 ± 7.8	4	0.387	8.3 ± 3.3	6.4 ± 2.8	7	0.031	0.042
MFR, %	8.1 ± 3.5	9.2 ± 3.5	4	0.434	7.2 ± 2.4	5.7 ± 2.2	7	0.028	0.037
HRF, %	3.0 ± 4.1	3.4 ± 4.6	4	0.381	1.1 ± 1.2	0.7 ± 0.6	7	0.286	0.236
MCH, pg/cell	29.6 ± 0.5	29.5 ± 0.8	4	0.921	30.3 ± 1.0	30.6 ± 1.1	7	0.281	0.542
MCHC, g/dL	33.7 ± 0.3	33.1 ± 0.4	4	0.061	33.5 ± 0.5	33.7 ± 1.0	7	0.518	0.066
MCV, fL	87.8 ± 1.6	89.2 ± 1.9	4	0.085	90.6 ± 3.0	90.9 ± 2.5	7	0.482	0.367
RBC, × 10^6^ µL^−1^	4.8 ± 0.3	5.0 ± 0.2	4	0.438	4.8 ± 0.2	4.9 ± 0.3	7	0.235	0.914
RET#, *n*	0.1 ± 0.0	0.1 ± 0.0	4	0.162	0.1 ± 0.0	0.1 ± 0.0	7	0.496	0.976
RET, %	1.7 ± 0.5	1.4 ± 0.4	4	0.091	1.4 ± 0.3	1.2 ± 0.4	7	0.308	0.893
OFF‐score	66.4 ± 13.0	76.7 ± 9.0	4	0.170	75.9 ± 10.3	85.6 ± 11.8	7	0.081	0.498
Androsterone, ng × mL^−1^	2447.9 ± 163.3	2400.9 ± 795.7	5	0.116	2589.6 ± 1264.0	2375.1 ± 1218.0	5	0.084	0.764
Etiocholanolone, ng × mL^−1^	2377.8 ± 1172.5	2552.3 ± 1358.0	5	0.491	2043.1 ± 973.8	2130.7 ± 1031.5	5	0.261	0.752
Epitestosterone, ng × mL^−1^	23.7 ± 15.3	22.2 ± 10.0	5	0.005	29.9 ± 27.2	26.7 ± 18.2	5	0.043	0.875
Testosterone, ng × mL^−1^	31.0 ± 26.6	25.5 ± 20.5	5	0.002	35.5 ± 32.8	32.4 ± 28.5	5	0.012	0.501
Testosterone/epitestosterone ratio	1.1 ± 1.0	1.0 ± 0.8	5	0.001	1.0 ± 0.8	1.2 ± 0.9	5	0.006	0.105
5α‐Androstane‐3α,17β‐diol, ng × mL^−1^	50.0 ± 19.4	36.7 ± 9.2	5	0.037	63.2 ± 39.9	48.5 ± 25.7	5	0.086	0.523
5β‐Androstane‐3α,17β‐diol, ng × mL^−1^	176.6 ± 186.7	130.2 ± 89.9	5	0.012	146.8 ± 114.3	162.0 ± 157.6	5	0.008	0.304

*Note*: Descriptive data are the mean (SD) for the following parameters: HRF, high fluorescence ratio; IRF, immature reticulocyte fraction; MCH, mean corpuscular haemoglobin; MCHC, mean corpuscular haemoglobin concentration; MCV, mean corpuscular volume; MFR, medium fluorescence ratio; OFF‐score calculated as ([Hb] × 10) − [60 × RET%]; RBC, red blood cell (count); RET#, reticulocyte count. The *p*‐values are presented indicating the time and treatment effects (INCO vs. AIR).

### 3.5 Effects of INCO on submaximal and maximal swimming performance

No significant time or treatment effects were observed in swimming speed corresponding to 2 and 4 mM of blood lactate, 200 m swimming time or average speed during the 200 m swimming test, indicating no effect of time or treatment (INCO vs. AIR) (Table 2).

## 4 DISCUSSION

The present findings are the first to show that administration of low doses of CO in the inspired air at rest and at sea level, mimicking the LHTL approach, elicits favourable adaptations in Hb_mass_, RBCV and BV compared with regular sea‐level training. In contrast, the muscle oxidative capacity of the triceps brachii muscle, in addition to submaximal and maximal swimming performance metrics during standard testing in the pool, were not affected. Notably, standard haematological markers used to interpret Hb_mass_ changes and the hormonal blood parameters were not significantly affected following CO supplementation. These findings are of interest because they might inform future research exploring potential applications within the context of the Athlete Biological Passport.

### 4.1 Haematological variables

The hypoxic stimulus induced by CO, although based on several assumptions as outlined in Section 2, was estimated to be 847.9 km h. For instance, this estimated hypoxic dose can be accumulated after 21 days at ∼2500 m a.s.l., using the LHTL protocol with 16 h per day spent at altitude.

Remarkably, Hb_mass_ increased by ∼6% following INCO compared with post‐AIR rather than the 3.4% predicted by the exponential equation derived from the meta‐analysis of altitude‐training studies by Garvican‐Lewis et al. (2016). However, the magnitude of the change in Hb_mass_ observed in the present study is not unprecedented; a similar response (between 5.5% and 8.6%) has been reported with a comparable hypoxic dose (i.e., 882 km h) using the LHTL method in elite race walkers (Saunders et al., 2010). For comparison, ∼6% increase in Hb_mass_ has been reported in swimmers exposed to greater hypoxic doses. Specifically, Wachsmuth et al. (2013) observed a 7.2% increase in Hb_mass_ with a hypoxic dose ranging from 1123.9 to 1445.0 km h, and Mujika et al. (2024) reported a 5.6% increase after 1177.4 km h (22 days at 2320 m a.s.l.).

The relationship between Hb_mass_ and ${\dot{V}}_{O_{2}\max}$ has been proposed to be a fundamental factor in enhancing endurance performance. Existing literature suggests that an increase of 1 g in Hb_mass_ corresponds to an increase in ${\dot{V}}_{O_{2}\max}$ by ∼4 mL min^−1^ (Schmidt & Prommer, 2010). Given that ${\dot{V}}_{O_{2}\max}$ was not measured in this study, we do not know the effect of increasing Hb_mass_ on ${\dot{V}}_{O_{2}\max}$. However, we can speculate that a rise of ∼54 g in Hb_mass_ post‐INCO would theoretically correspond to an increase in ${\dot{V}}_{O_{2}\max}$ of ∼216 mL min^−1^, which might be meaningful in the perspective of elite endurance athletes.

This study demonstrated that periodic exposure to a low dose of CO designed to mimic the LHTL approach promotes haematological adaptations of a magnitude similar to that reported in literature following a similar hypoxic dose, i.e., 3–4 weeks training camp at moderate altitude. Although erythopoietin levels were not measured in the present study, previous research has shown that CO acutely induces a reduction in arterial oxygen content and an upregulation of erythopoietin comparable to that observed in normobaric hypoxia (Montero & Lundby, 2019). This upregulation of erythopoietin is likely to be the main stimulus driving the increase in Hb_mass_, as demonstrated following chronic CO supplementation (Schmidt et al., 2020).

In contrast to the haemoconcentration and PV reductions commonly reported after exposure to normobaric or hypobaric hypoxia, caused by a fluid shift from the intra‐ to extravascular space rather than fluid loss (Siebenmann et al., 2024), PV in the present study was not affected by CO‐induced hypoxia. Although CO mimics hypoxic conditions (Richardson et al., 2002), it does not elicit the hypoxia‐induced tachycardia or ventilatory response (Hanada et al., 2003), which probably contributed to the preservation of PV observed here.

### 4.2 Submaximal and maximal performance variables

In the present study, we selected the blood lactate profile test, which is correlated well with endurance performance and is widely used in practice (Faude et al., 2009), as a measure to assess the effect of the hypoxic dose induced by CO on endurance performance. Speed at 4 mM in blood latacte has been proposed as a valid performance marker in endurance sports (Jacobs et al., 2011). In addition to enhancing the potential for oxygen delivery to working muscles, increased Hb_mass_ can also improve the buffering capacity of the blood for pH by facilitating the transport of carbon dioxide and binding with H^+^ ions (Mairbaurl, 2013), thereby potentially improving performance. In our study, no significant differences in standardized submaximal and maximal swimming performance were reported following INCO compared with sea‐level training. The absence of changes in performance in the present study might be related, in part, to the nature of the tests used, because submaximal workloads and a 200 m maximal effort might have been too short in duration to capture potential benefits of increased blood O_2_‐carrying capacity. In addition, the timing of post‐intervention testing might not have been optimal, because no dedicated tapering phase was implemented, which does not reflect the common practice of applying tailored tapering strategies prior to competition (Mujika et al., 2019). Similar observations have been made in previous work on competitive swimmers, where improvements in performance following altitude exposure were small, non‐significant or transient, even with an increased Hb_mass_. For instance, after a hypoxic dose of ∼1100 km h using either natural LHTH or simulated LHTL, swimming performance in 26 elite swimmers was unchanged or slightly impaired 1 day (0.4% ± 0.4%) and 7 days (−0.2% ± 0.7%) post‐camp, with no difference from pre‐camp levels at 14 days (−0.3% ± 0.8%) and 28 days (0.2% ± 0.9%) (Gough et al., 2012). Likewise, no significant sea‐level performance changes were observed immediately after 1169 km h (LHTH, 3 weeks at 2320 m) (Astridge et al., 2024), and Wachsmuth et al. (2013) reported either a negative trend or no improvement until ∼14 days post‐camp (1123.9–1445.0 km h). Collectively, these findings indicate that swimming performance often shows little or no improvement, and might even decline, immediately after returning to sea level from high‐altitude camps, with a potential ‘window’ of 3–4 weeks required before performance gains become evident. One potential explanation of little or no improvement in swimming performance in the first days post‐altitude camp might involve a transient reduction in buffering capacity, resulting from hypoxia‐induced suppression of both bicarbonate and non‐bicarbonate buffering systems (Böning et al., 2001). Additionally, the re‐adaptation of various endocrine systems, such as those regulating aldosterone and erythropoietin, might contribute to these delayed responses (Wachsmuth et al., 2013).

### 4.3 Muscle oxidative capacity detected by NIRS

Although it was not possible to obtain a skeletal muscle biopsy, information about skeletal muscle oxidative capacity was estimated by using a new approach recently proposed by our groups (Pilotto et al., 2022) through a non‐invasive method by NIRS. Over the past decades, NIRS has been used to evaluate different aspects related to muscle physiology (for a review, see Barstow, 2019). More closely related to muscle oxidative metabolism, previous studies evaluated fractional O_2_ extraction during exercise (Boone et al., 2016; Porcelli et al., 2019). However, these approaches primarily reflect the balance between oxygen delivery and extraction rather than measuring muscle oxidative capacity itself. In our protocol, we overcame this limitation because we evaluated muscle oxidative capacity independently from oxygen delivery. Indeed, changes in the relative concentration of oxy‐ and deoxyhaemoglobin and myoglobin (O_2_HbMb + HHbMb) were determined during an ischaemic phase (i.e., independently from oxygen delivery). More specifically, the recovery rate constant (*k*) of muscle oxygen uptake was established from the rate of decline in muscle tissue saturation index [TSI = O_2_HbMb/(O_2_HbMb + HHbMb)] under serial, intermittent arterial occlusions. This approach has been shown to correlate well with mitochondrial respiration measured *ex vivo* in permeabilized muscle fibres by high‐resolution respirometry (Pilotto et al., 2022; Ryan et al., 2014), and it has recently been demonstrated to be able to differentiate between swimmers of varying tiers and is positively associated with both training volume and performance (Villanova et al., 2025).

In the present study, muscle oxidative capacity was not influenced by CO exposure and did not differ pre to post each block (no effect of time). This contrasts with our initial hypothesis that CO inhalation would promote skeletal muscle adaptations, such as markers of mitochondrial and capillary density (Pecorella et al., 2015). The unchanged skeletal muscle oxidative capacity is consistent with previous studies on altitude training. For instance, Jacobs et al. (2013) reported no changes in mitochondrial function after 9–11 days of exposure to 4559 m a.s.l. Likewise, different aspects of mitochondrial oxidative capacity were unaffected after 6 weeks of normobaric hypoxic training (equivalent to an altitude of 2500 m a.s.l.) in moderately trained individuals (Robach et al., 2014). Therefore, given that non‐invasive estimates of skeletal muscle oxidative capacity showed no effect from CO, we interpret CO as not having a direct or indirect (i.e., CO‐induced hypoxia) large impact on skeletal muscle of trained individuals as previously proposed by Richardson et al. (2002).

### 4.4 Haematological markers

No CO‐induced effect on standard haematological markers used to interpret changes in Hb_mass_ was noted, except for a minor decline in IRF. This finding is opposite to a previous study, in which IRF increased after 1 week of daily CO administration, returning, and stayed at baseline levels during the remaining 2 of the total 3 weeks intervention (Schmidt et al., 2020). The reason behind the decrease in IRF is not known but might indicate that erythropoiesis is beginning to slow down at the end of the training block. It could also be a coincidental finding, given that IRF and medium fluorescence ratio (MRF) WHO guidelines show natural large intra‐individual variations (Krumm et al., 2024). A limitation with our study design is that the haematological markers could only be compared statistically before and after intervention and thus do not mimic the Athlete Biological Passport approach, whereby the results are monitored longitudinally. Future studies should involve regular monitoring of Athlete Biological Passport parameters before, during and after carbon CO exposure to assess more carefully the haematological profile in response to repeated CO use.

In addition to haematological parameters, the urinary androgen markers included in the Athlete Biological Passport were determined, showing no differences between the groups. The effect of CO on the urinary steroid profile has not been studied before to our knowledge. A notably finding was that some steroids displayed higher concentrations pre‐sampling than after 4 weeks of intensified training regardless of treatment. An increased training load has been associated with lower urinary androgen levels (Eklund et al., 2021). An additional Athlete Biological Passport module including serum testosterone and androstenedione has recently been implemented in anti‐doping testing. Hypoxia induced by residing at altitudes has been associated with decreased serum testosterone (Mujika et al., 2019). Unfortunately, neither serum nor plasma was available for steroid analyses in this project, hence it was not possible to evaluate the hormonal status of the participants. Conclusively, the haematological and urinary steroid parameters were not affected by CO supplementation and the administration protocol used herein.

### 4.5 Limitations

Our study is not without limitations that should be acknowledged. Although both men and women were included, the small sample size of eight participants might limit the generalizability of the findings to a broader population. However, the crossover design used requires a smaller sample size than other designs, such as parallel groups (Grenet et al., 2020). Another major limitation is the inability to explain the mechanism by which CO supplementation induced the observed erythropoietic response. Additionally, the lack of skeletal muscle biopsy raises questions about the specific mechanisms and potential structural and functional adaptations in response to CO inhalation compared with sea‐level training. Furthermore, the absence of clear changes in performance might relate, in part, to the choice of performance tests and the timing of post‐intervention testing, because no tailored tapering phase was implemented. Finally, the inclusion of only one baseline sample limited the possibility to assess the full Athlete Biological Passport adaptive model.

### 4.6 Ethical aspects

The present study, along with the recently published work from our group (Urianstad et al., 2024), represents the first and only evidence in well‐trained individuals to indicate physiological benefits of low‐dose CO inhalation. From January 2026, the non‐diagnostic use of CO through rebreathing systems or any equipment designed to deliver CO is explicitly prohibited by the World Anti‐Doping Agency (WADA, 2025). This update is fully consistent with the findings of the present study, which demonstrate measurable physiological effects of repeated low‐dose CO inhalation. Consequently, the question is no longer whether this method should be considered permissible, but rather how to detect its potential misuse among athletes under anti‐doping regulations. The prohibition aligns with growing health and ethical concerns regarding repeated CO inhalation, which had already led the Union Cycliste Internationale (UCI, 2025) to impose a ban owing to potential performance‐enhancing and safety risks. Our study was conducted before this prohibition came into effect and aimed solely to investigate the physiological mechanisms underlying CO‐induced erythropoietic and circulatory adaptations.

The beneficial (Millet & Brocherie, 2020) or non‐beneficial effects of hypoxic training (Siebenmann & Dempsey, 2020) in elite athletes have been debated widely. The aim of the present study was to test the hypothesis that blindly inhaling low doses of CO while residing at sea level to simulate the popular LHTL strategy confers physiological benefits in comparison to sea‐level training. This study reinforces evidence in favour of the hypoxic effect on Hb_mass_, RBCV and BV, which cannot be attributed to a placebo effect, because the participants were blinded to the intervention and owing to the cross‐over study design. However, performance metrics were unaffected by the hypoxic dose, in line with the few blinded placebo‐controlled studies (Saunders et al., 2010; Siebenmann et al., 2012).

Importantly, this proof‐of‐concept study was conducted for scientific purposes only and is not intended to promote or endorse the use of CO among athletes or the general public. Although CO has been explored as a therapeutic agent for its signalling properties in the past decade (Bansal et al., 2024), it is crucial to highlight that voluntary CO exposure without expertise and appropriate equipment (e.g. calibrated gas analysers, CO detectors and uncontaminated CO sources), is dangerous and can result in serious acute and chronic health problems or death (WHO guidelines 2010). We therefore discourage any attempt at voluntary CO exposure for any purpose.

## 5 CONCLUSION

In conclusion, these findings suggest that periodic CO inhalation at sea level can mimic some haematological adaptations of the LHTL approach, significantly increasing Hb_mass_ and RBCV to levels comparable to those reported after 3–4 weeks of moderate‐altitude training, but these adaptations did not translate into improved performance. Exploratory haematological markers related to anti‐doping monitoring were largely unaffected.

From January 2026, the non‐diagnostic use of CO through rebreathing systems or other delivery equipment is explicitly prohibited by the World Anti‐Doping Agency, consistent with the physiological effects observed in the present study. The focus should now shift towards improving methods to detect and prevent potential misuse among athletes. We emphasize that this work is intended solely for the scientific community and that voluntary CO exposure for performance enhancement is unsafe and prohibited under WADA regulations.

## AUTHOR CONTRIBUTIONS

Daniele A. Cardinale conceived and designed the research, performed statistical analyses and prepared figures; Daniele A. Cardinale, Simone Villanova and Lena Ekström performed experiments; Daniele A. Cardinale, Simone Villanova, Lena Ekström and Simone Porcelli interpreted results of experiments; Daniele A. Cardinale and Simone Villanova drafted the manuscript. All authors revised and approved the final version of the manuscript and agree to be accountable for all aspects of the work in ensuring that questions related to the accuracy or integrity of any part of the work are appropriately investigated and resolved. All persons designated as authors qualify for authorship, and all those who qualify for authorship are listed.

## CONFLICT OF INTEREST

The authors declare that the research was conducted in the absence of any commercial or financial relationships that could be construed as a potential conflict of interest.

## Data Availability

All data are available on https://figshare.com/s/b9f2088baf3af9e01f00.
